# Perspectives on quality mental health care from Brazilian and Cape Verdean outpatients: Implications for effective patient-centered policies and models of care

**DOI:** 10.3402/qhw.v9.22839

**Published:** 2014-01-21

**Authors:** Maria De Jesus, Tara R. Earl

**Affiliations:** 1 School of International Service, Center on Health, Risk, and Society, American University, Washington, DC, USA; 2 ICF International, Inc., Division of Public Health and Survey Research, Atlanta, GA, USA; 3 Cambridge Health Alliance, Center for Multicultural Mental Health Research, Harvard Medical School, MA, USA

**Keywords:** Patient perspectives, quality of care, mental health, Brazilian, Cape Verdean, patient-centered care

## Abstract

Mental health providers are increasingly coming into contact with large and growing multi-racial/ethnic and immigrant patient populations in the United States. Knowledge of patient perspectives on what constitutes quality mental health care is necessary for these providers. The aim of this study was to identify indicators of quality of mental health care that matter most to two underrepresented immigrant patient groups of Portuguese background: Brazilians and Cape Verdeans. A qualitative design was adopted using focus group discussions. Six focus groups of patients (n=24 Brazilians; n=24 Cape Verdeans) who received outpatient mental health treatment through public safety net clinics in the northeast region of the United States were conducted. The Consensual Qualitative Research analytic method allowed us to identify three quality of care domains: *provider performance*, *aspects of mental health care environment*, and *effectiveness of mental health care treatment*. Provider performance was associated with five categories: relational, communication, linguistic, cultural, and technical competencies. Aspects of mental health care environment were linked to two categories: psychosocial and physical environment. Effectiveness of mental health care treatment was related to two categories: therapeutic relationship and treatment outcomes. Study findings provide useful data for the development of more culturally appropriate and effective patient-centered models and policies in mental health care.

Immigrants (referring to foreign-born individuals and therefore not U.S. citizens at birth) represent nearly 13% of the U.S. population, and this rapidly growing segment of the population is mainly of non-European origin (U.S. Census Bureau, 2012). Given these demographic trends, mental health professionals are increasingly coming into contact with diverse immigrant patient populations and are challenged to provide patient-centered quality of care (QOC). Clinicians often lack sufficient knowledge about which attributes of QOC matter the most to their patients, the absence of which contributes to poor clinical encounters and potentially unfavorable treatment outcomes (Alegria, Canino, et al., 2008; Alegria, Chatterji, et al., 2008; Smedley, Stith, & Nelson, 2003). Moreover, many mental health care service delivery models have evolved without considering the perspectives of immigrant patient populations in any systematic manner, which poses additional treatment barriers for these populations in terms of QOC issues (Bettancourt, Green, Carrillo, & Ananeh-Firempong, 2003).

The Institute of Medicine defines QOC as: “the degree to which health services for individuals and populations increase the likelihood of desired health outcomes and are consistent with current professional knowledge” (Lohr & Committee to Design a Strategy for Quality Review and Assurance in Medicare, 1990). However, scientific evidence suggests that patients may not conceptualize QOC in the same way as health care providers (Donabedian, 1980, 1988; Earl, Alegria, Mendieta, & Linhart, 2011; Pope-Davis et al., 2002; Tucker et al., 2007). Moreover, QOC has most meaning when applied to individual users given that they are the recipients of care (Campbell, Rowland, & Buetow, 2000; Sixma, Kerssens, van Campen, & Peters, 1998; Sofaer, & Firminger, 2005; Stichler & Weiss, 2000).

Numerous studies have examined and measured patients’ experiences, expectations, and satisfaction with their health care environment (Bowling et al., 2012; Browall, Koinberg, Falk, & Wijk 2013; Grundberg, Ebbeskog, Dahlgren, & Religa, 2012; Martinsson, Fagerberg, Lindholm, & Wiklund-Gustin, 2012; Martinsson, Wiklund-Gustin, Lindholm, & Fagerberg, 2011; Rasmussen & Hellzen, 2013). Many have specifically examined relationship qualities important in provider–patient interactions across the four most recognized racial and ethnic groups in the United States: African Americans, American Indians and Alaskan Natives, Asian and Pacific Islanders, and Hispanic/Latinos (Buck & Alexander, 2006; Kirsh & Tate, 2006; Tunner & Salzer, 2006; Ware, Tugenberg, & Dickey, 2004). However, these studies have not examined the perceptions of QOC of specific underrepresented immigrant patient populations, even though evidence suggests that immigrant patients have different preferences for mental health care than aggregate-level racial/ethnic groups (Pumariega, Rothe, & Pumariega, 2005).

The current study fills a gap in the literature by examining perceptions of QOC within a sample of two underrepresented immigrant patient groups of Portuguese background: Brazilian and Cape Verdean immigrants who receive mental health treatment through public safety net clinics in the northeast region of the United States. These immigrant groups are often grouped in aggregate-level racial/ethnic groups based on U.S. Census data. For example, Brazilians and Cape Verdeans are typically grouped in studies of Latinos or non-Latino Whites, even though these groups have distinct cultural values, beliefs, and norms that differ from the respective majority groups (Flores, 2005). More recently, the Institute of Medicine (2009) has recommended the use of granular ethnicity, which considers a more in-depth understanding of an individual's ethnic origin and heritage for improving the quality of health care. These data are increasingly important for clinicians, administrators, and policymakers in mental health systems to develop more culturally appropriate and effective models and policies of mental health care service delivery (Bettancourt et al., 2003).

## Brazilian and Cape Verdean immigrants in the United States

Brazilians and Cape Verdeans comprise two immigrant groups with a common Portuguese background who, despite their growing numbers, are underrepresented in the mental health literature. According to the 2006–2010 American Community Survey, there are 347,346 Brazilians in the United States, with nearly half of all Brazilian immigrants living in the northeastern United States, primarily in the states of Massachusetts, New York, and New Jersey (Lima & Siqueira, 2007). Massachusetts alone is home to 65,170 Brazilians, approximately 19% of the national Brazilian population (Lima & Siqueira, 2007).

In terms of the Cape Verdean population, there are 92,936 Cape Verdeans in the United States, with more than half residing in Massachusetts (American Community Survey, 2011). Moreover, the number of individuals with Cape Verdean ancestry who are living in the United States, including both migrants and their descendants, is higher than in any other country (Carling, 2002).

## Aim

The aim of this study was to define indicators of “good” quality care from the perspective of Brazilian and Cape Verdean immigrant patients.

## Methods

### Design

We adopted a qualitative design using focus group discussions. Focus groups are particularly useful for exploring several participants’ points of view, knowledge, and experiences, while also allowing them to raise questions and exchange ideas on a specific topic (Kitzinger, 1995). This method allowed us to capitalize on the group dynamics and make timely observations from the open communication among several participants (Kitzinger, 1995).

### Participants and setting

Participants were recruited from two outpatient safety net clinics in the northeast region of the United States. Each clinic was located within an urban community setting that was mostly populated by the respective immigrant populations. Eligibility criteria included: (1) at least 18 years of age; (2) have a diagnosis of depression (i.e., major depressive disorder, depressive disorder Not Otherwise Specified (NOS), or dysthymic disorder); (3) have been seen at one of the outpatient clinics no more than three times; (4) self-identified as Brazilian or Cape Verdean.

### Data collection

This study presents data from six in-depth focus group discussions with patients who self-identified as Brazilian (n=24; three focus groups) or Cape Verdean (n=24; three focus groups). First, a capacity-to-consent screen was administered in each participant's preferred language to ensure that the individual was able to adequately consent to the study (Zayas, Cabassa, & Perez, 2005). All participants were deemed capable of consenting. Once consent was obtained, participants were asked to complete a brief sociodemographic questionnaire that assessed items such as gender, age, ethnicity, country of birth, years of education, annual household income, and whether they received prior mental health treatment. The questionnaire was available in English, Portuguese, or Cape Verdean Creole.

Subsequently, focus groups were facilitated by a trained, English-speaking moderator and a professionally trained interpreter, who spoke in the participants’ native language. A semi-structured focus group discussion guide was developed to ensure consistency during each focus group session. Participants were asked 14 open-ended questions aimed at eliciting their perceptions of quality mental health care. Sample questions included: How do you know when you receive good quality mental health care? How do you know when you receive poor quality mental health care? and When looking for a provider what are some of the characteristics you seek?

Each session lasted between 60 to 90 min and was held at each outpatient clinic. Participants received a $20 gift certificate to Target at the end of each focus group session. All data were professionally transcribed and translated into English.

### Ethical considerations

All participants received a letter describing the purpose and procedure of the study. The letter also stated that their participation was voluntary, that the information would be kept confidential, and that their contributions would be unidentifiable in the final report. Approval for the study was obtained from the Boston College Institutional Review Board and each of the outpatient clinics.

### Data analysis

Our data analytic strategy of choice was the Consensual Qualitative Research (CQR) method (Hill, Thompson, & Williams, 1997; Hill et al., 2005). CQR incorporates elements from phenomenology (Giorgi, 1985), grounded theory (Strauss & Corbin, 1998), and comprehensive process analysis (Elliott, 1989). From these qualitative approaches, there is an emphasis on consensus among judges to construct findings and the use of words rather than numbers to reflect meaning in the data (Hill et al., 2005). In terms of a philosophical stance, CQR is predominantly constructivist, with some postpositivistic elements. A major assumption of CQR is that individuals construct their reality and that there are multiple, equally valid, socially constructed versions of “the truth.” We chose this rigorous method because it was suitable for our aim to explore commonalities in experiences and perceptions of quality mental health care among a small sample of participants (Hill et al., 2005). To attain consensus, the CQR method demands that the team members discuss disagreements, a process which helped us to unravel the complexities and ambiguities of the data. We followed the three key steps of CQR to analyze the data (Hill et al., 2005):

1. *Coding into Domains*. After reviewing each focus group transcript, the first step was to identify the overarching domains (i.e., topic areas). Following the CQR method, the second author acted as a level one auditor by reading each focus group transcript and asking the research assistants to independently identify domains. In this role, she reviewed and judged the domains based on how well they reflected the content of the transcripts. Subsequently, a research team meeting was held to discuss, reduce, modify and come to consensus on the emerging domains.
2. Abstracting *Core Ideas within Domains*. During the next stage of analysis, core ideas for each domain were abstracted across each transcript. This involved examining the participants’ words and reducing them into meaning units, representing the essence of each statement. To minimize potential bias, a second level external auditor was invited to independently review the coded data and provide feedback. Suggestions from the auditor included general observations about emerging domains or possible subcategories within the domains. The external auditor's comments were shared with the team and discussed until consensus was achieved.
3. *Completing Cross-Analysis*. We conducted a cross-analysis to identify common and different themes reflected in the core ideas across each ethnic group. These themes were organized into domains, categories, and subcategories. NVivo 8 qualitative research software was used for data analysis. We also conducted member checking, a method suggested by Miles and Huberman (1994) to assess the validity of the analysis. The results were reviewed by a panel of six study participants who had voluntarily consented to be contacted for feedback. The participants agreed with the established domains, categories, and subcategories.

## Results

### Sample characteristics

Most Cape Verdean participants were female (75%), between the ages of 35 to 64 (89%), and were, on average, 34 years old when they moved to the United States. Brazilian participants were split in half in terms of gender and were mostly between the ages of 35 to 64 (86%). They were on average 31 years old when they moved to the United States. All Cape Verdean participants reported earning less than $35,000 per year, while the annual household income for the Brazilian participants was more disparate, ranging from less than $15,000 per year to $75,000. Brazilian and Cape Verdean participants also differed in terms of education level. Most of the Brazilian participants (87%) had 16 years or more of education, while the majority of Cape Verdeans had 11 years of education or less (89%). Eighty-two per cent of the total sample indicated a history of receiving mental health treatment.

### Domains, categories, and subcategories

Analysis of the focus group transcripts formed a structure comprising three primary domains of quality mental health care: (1) *provider performance*, (2) *aspects of mental health care system, and* (3) *effectiveness of mental health care treatment*. These domains together with their categories and subcategories are illustrated with quotations from the focus group discussions and presented in this section. Table I presents a summary of primary domains, categories, and subcategories.

**Table I:** Primary domains, categories, and subcategories of perceived quality of care (QOC) in mental health from the perspectives of Brazilian and Cape Verdean patients

Primary domains	Categories	Subcategories
Provider performance	Relational competencies	Provider is attentive
		Provider is available to patient
		Provider is respectful toward patient
		Provider is nonjudgmental
		Provider is thoughtful
		Provider is relatable
		Provider encourages patient
		Provider is egalitarian with patient
	Communication competencies	Provider communicates in a comprehensible manner with patient
		Provider conveys comforting/acceptable nonverbal cues toward patient
		Provider explains instructions clearly to patient
	Linguistic competencies	Provider can speak and understand patient's native language
	Cultural competencies	Provider is of similar cultural background as patient
		Provider is familiar with patient's cultural background
		Provider is aware of cultural differences
	Technical competencies	Provider is thorough with patient's treatment
		Provider is well-trained
Aspects of mental health care environment	Psychosocial environment	All mental health care staff and team members have good professional attitude and conduct
	Physical environment	Mental health care clinic/hospital has good resources, including advanced technology
Effectiveness of mental health care treatment	Therapeutic relationship	Productive interactions with provider
	Treatment outcomes	Symptoms improve over time

## Provider performance

Overwhelmingly, Brazilian and Cape Verdean participants agreed that the onus is on the mental health professional to provide QOC. As a Cape Verdean participant expressed: “Good quality mental health care is when the provider is tending to me well.” This domain comprises five categories:

### Relational competencies

The first category refers to provider characteristics that are perceived to be helpful in fostering a relationship with the patient. Eight associated subcategories are described below:

#### Provider is attentive

Most participants shared that a provider “who listens and captures everything” is an important aspect of QOC.

#### Provider is available to patients

Having a mental health provider be there when you need him/her also reflects QOC for both Brazilian and Cape Verdean participants. For example, one Brazilian participant recounted: “When you need [someone] and he's there to see you, that's a sign of good quality as opposed to when you call and talk to the answering machine…” Cape Verdean participants also agreed: “I work weekdays until 6:00 pm. If the doctor is available during evening hours like 7 pm or weekend hours like 9 am to 1 pm, that is good.”

#### Provider is respectful toward patient

Several participants agreed that another indicator of QOC is when the mental health care provider conveys respect toward patients. As a Brazilian participant aptly stated:

I would say that a doctor who sees you as a regular patient and does not assume that because you're Brazilian, you are more stupid than other cultures. Just because you don't speak the language or you chose him, it doesn't mean you can't communicate with anybody else … That's where I think it can be disrespectful: for the doctor to look down to you. That's something he should know, that I'm his patient and I deserve as much respect as an American citizen.

#### Provider is nonjudgmental

Most Brazilian and Cape Verdean participants also thought quality mental health care means being able to talk with a professional without being negatively judged. As a Brazilian participant stated: “A professional listens to you and does not judge you. It's not like your friend who's going to criticize you.”

#### Provider is thoughtful

In addition, several participants agreed that QOC is when a mental health provider thinks carefully before he/she speaks to the patient. One Brazilian participant contrasted his experiences with mental health care in the United States and Brazil: “My doctor, he would stop, think of the word, before he just let it out. He came out with the right words, and that's where you feel the difference [compared to Brazil] where the doctor speaks without first thinking carefully about how to express him/herself.”

#### Provider is relatable

Brazilian and Cape Verdean participants also indicated that part of QOC is when a mental health provider shows some of their own imperfections, as this makes them more relatable to patients. As one Cape Verdean participant stated: “Sometimes I also see that the doctor seems to be having issues [he] isn't in a great mood, for example, so you're consoled in that way.”

#### Provider encourages patient

Many participants further described QOC as having a mental health provider who is supportive, especially given that it is viewed as embarrassing and/or shameful to seek mental health care services in the given cultures. As one Cape Verdean participant recounted: “Even when I did start coming for treatment it was still embarrassing to speak to a doctor about it and the doctor was really good and said I shouldn't be embarrassed. She encouraged me to keep coming.”

#### Provider is egalitarian with patient

Most participants also felt that it is important that their mental health provider treats them as “equals.” As one of the Cape Verdean participants shared: “We talk on equal grounds to the point where the doctor shares [some of] her experiences with me, just like any other person would.”

### Communication competencies

This second category includes three subcategories:

#### Provider communicates in a comprehensible manner with patient

Several Brazilian and Cape Verdean participants mentioned that QOC entails a provider speaking to them in a manner that they could easily understand. As a Cape Verdean participant recounted: “Sometimes even with native Cape Verdean doctors who speak Kriolu, it is difficult for someone without the knowledge to understand them. It is good when they are able to break down the language for us.”

#### Provider conveys comforting/acceptable nonverbal cues toward patient

Participants shared that QOC also involves a provider using nonverbal cues such as physical touch to comfort the patient. As a Cape Verdean participant stated: “When I told her about my problems, she ran her hand down my back while telling me that I will eventually regain control. This consoled me a lot.” A few Brazilians also mentioned that eye contact from the provider is very important to engage the patient in therapy.

#### Provider explains instructions clearly to patient

This subcategory emerged for both Brazilian and Cape Verdean participants. As a Cape Verdean described: “Good quality is having the explanation be clear about what I should do and what I shouldn't do …”

### Linguistic competencies

One subcategory is associated with this third category:

#### Provider can speak and understand patient's native language

This was perceived as a key aspect in defining quality of mental health care for both Brazilian and Cape Verdean participants. One Brazilian participant who traveled 40 min to get to the clinic remarked: “I come here just because my doctor can speak Portuguese and I feel more comfortable.” Other participants agreed. Most Cape Verdean participants in the group did not speak Portuguese and shared that they felt more comfortable speaking in Kriolu and having access to a Kriolu-speaking provider. This meant that patients could talk and be understood directly by their provider, and this was preferable to having an interpreter. Some Cape Verdean participants shared experiences of incompetent interpreters who do not translate exactly what the patient has stated.

### Cultural competencies

Although all participants mentioned that linguistic compatibility with a provider is important for QOC, they also stated that it goes beyond language and that “culture makes a difference.” This fourth category includes three subcategories:

#### Provider is of similar cultural background as patient

There were differences among participants in terms of whether QOC means having a provider of the same cultural background. When asked further about whether language compatibility between patient and mental health care provider is sufficient for good QOC, some participants felt that this was not enough. Although all participants acknowledged that the language barrier is important to overcome, some also felt that having a similar cultural background is important to receive good quality care, while others did not. As one Brazilian stated: “By having someone who is from your country, it's easier for them to detect the problem because they know exactly where you come from and how you've been raised …” Similarly, some Cape Verdean participants felt that a provider's cultural background matters and that “the obstacle [to getting good quality treatment] is not having a Cape Verdean doctor.”

Others did not feel that a mental health provider who has a similar cultural background is a necessary aspect for good QOC. As mentioned by a Brazilian participant: “For me, there's no difference. I was cared for by a Brazilian doctor and an American doctor, and I don't think there was a difference.” Similarly, a Cape Verdean participant stated: “I don't think he [a Cape Verdean mental health provider] will understand me better than a non-Cape Verdean doctor would.”

#### Provider is familiar with patient's cultural background

Although there was no consensus among participants about whether a mental health provider needs to be of a similar cultural background as the patient, all participants did agree that QOC could only be achieved if a mental health care provider is familiar with a patient's culture and has a shared cultural understanding. As one Brazilian respondent remarked: “I think there's something important about having a certain level of knowledge about our culture by the mere fact that you're working with the mind.”

#### Provider is aware of cultural differences

This subcategory was more common for Brazilian than for Cape Verdean participants. As one Brazilian participant noted: “I think that culture makes a difference because Brazilians have a culture that is different from that of the Portuguese and that of the Cape Verdeans. The doctor needs to know this.” Some Cape Verdeans also expressed similar thoughts.

### Technical competencies

Most participants mentioned that a mental health provider's credentials are important for QOC. As described by a Brazilian participant: “If she [mental health provider] was approved by the board to work here and she is a provider of psychology, then I am not worried. I would be confident in her technical abilities.” Two subcategories are associated with this fifth category:

#### Provider is thorough with patient's treatment

Several Brazilian and Cape Verdean participants mentioned how they like that U.S. mental health care providers are very thorough in their treatment compared to providers in their home country, and they perceive this as a key indicator of QOC. For example, Brazilian participants liked that U.S. mental health providers ask in-depth questions about the patient's personal and family history and compared it to their experience in Brazil where they felt that mental health care providers only scratch the surface with the types of questions they pose. As a Brazilian participant recounted: “In my treatment where I saw the difference is that there [in Brazil], when I went to the doctor, when she interviewed me, she did not ask me many questions about my past. This doctor here [in the United States] asked me about everything: my relationships, my home, my dad, my mom, my family, and my marriage.” Similarly, a Cape Verdean participant noted: “The moment you come to the doctor, the doctor asks you all about your problems. They also ask about your family relationships. I see that the doctor wants to help me with everything she can … I think that this is good treatment.”

#### Provider is well-trained

Many participants also stated that it is important for a mental health provider be properly trained and “have the appropriate background” to provide quality mental health care. As a Brazilian participant described: “She [mental health provider] had gone to medical school and she is very up-to-date on the tools that the scientific world makes available for her to help me. So I'm calm. When I sit here, I don't even know who the provider is, but I already know that she has the background to be sitting here.”

## Aspects of mental health care environment

When asked what encompasses QOC, many Brazilian and Cape Verdeans stated aspects of the mental health care environment, which was the second domain. This domain includes two categories:

### Psychosocial environment

This category covers patient perceptions of how well the mental health care team members interact with the patients.

#### All mental health care staff and team members have good professional attitude and conduct

Being well-received by clinical staff members and not just by their mental health providers seems to influence many of the participants’ perceptions of good quality mental health care. Many participants perceived the entire U.S. mental health care team as more professional than that of their country of origin. A Brazilian participant shared an example of a providers’ lack of professionalism in Brazil: “I was seeing the same professional as my wife and this professional told my wife things I had talked to her about in my session…She cut the bond of trust.” Another Brazilian participant also contrasted the U.S. and Brazilian mental health systems: “Over there [in Brazil], the nurses, you know, they don't care. In the U.S., the nurses care for us with warmth and attention. I think this is fantastic. I get sad because in our country, at least in São Paulo, where I live, it's very sad. The hospitals, the mental health care there is bad… Here in the U.S., the clinical and psychiatric system is fantastic.” Another Brazilian participant cautioned that one cannot make broad generalizations about QOC in the United States based on experiences in one state: “You're mentioning the United States, but it's not all over the United States that they have this care that they have here [in Massachusetts]. In Florida, it's horrible.”

### Physical environment

Patients also discussed physical aspects of the mental health care facility that they think are important in contributing to the quality of mental health care.

#### Mental health care clinic/hospital has good resources, including advanced technology

Participants contrasted a context rich in resources in the U.S. mental health system with that of Brazil: “The resources and technology at the hospital and clinics, those count for a lot, too. You can't compare. Brazil is a third world country and America, you know, is a first world country.”

## Effectiveness of mental health care treatment

All participants agreed that QOC directly links to the effectiveness of their mental health care treatment. This third domain consists of two categories, each of which is associated with one subcategory.

### Therapeutic relationship

#### Productive interactions with provider

Participants primarily assessed treatment effectiveness by whether they felt that the relationship with their provider was going well: “It's like a marriage. If you're with that person and the person doesn't respond to you, well, you let go of them!”

### Treatment outcomes

#### Symptoms improve over time

In addition, participants assessed mental health treatment effectiveness by whether they viewed their symptoms as getting better. As aptly described by a Cape Verdean participant: “For me after coming to this doctor, I feel much more relieved. Before I came here, my disorientation would happen to me three or four times a day. But now after doing this treatment, it only happens to me twice a day. I have received help here and I feel like it is better.”

## Discussion

The purpose of this study was to understand and describe QOC in mental health care through the eyes of Brazilian and Cape Verdean patients. The perspectives of these patients can provide a framework for understanding their unique mental health care preferences, which can improve mental health care service delivery for these populations (Bettancourt et al., 2003; Donabedian, 1988). Consistent with the literature, including Donabedian's (1988) structure–process–outcome model for improving health care quality, both Brazilian and Cape Verdean participants viewed provider performance as a key aspect defining QOC (Donabedian, 1980, 1988; Jun, Peterson, Zsidisin, 1998; Sofaer & Firminger, 2005; Stichler & Weiss, 2000). Both Brazilian and Cape Verdean participants viewed interpersonal performance and technical performance of the provider as relevant for QOC, despite socioeconomic and educational differences, with Brazilians representing higher levels on both criteria compared to Cape Verdeans. This finding is most likely related to the fact that the majority of participants, irrespective of socioeconomic and educational differences, shared a history of having received mental health treatment, which they likely drew on to contextualize their perspectives on quality mental health care.

Most Brazilians and Cape Verdeans also viewed language compatibility between provider and patient as extremely important. Additionally, most participants thought it was more important for the provider to have a shared understanding and familiarity with the patient's culture than it was for the provider to be of the same cultural background as the patients. These participants also valued the ways in which practitioners stepped outside of the professional constraints of their role and spoke to the patient on more equal terms. This finding is consistent with other studies on QOC (e.g., Ware et al., 2004). Furthermore, our findings on the assessment of effectiveness of mental health care treatment for Brazilians and Cape Verdeans are consistent with previous studies which also demonstrate that effective mental health treatment depends upon good communication and productive interactions between patients and mental health providers (Wills & Holmes-Rovner, 2006).

This study has some limitations. First, all of the focus groups were conducted in health care organizations that mostly serve low-income individuals; therefore some of our findings may not reflect the perspectives of Brazilian and Cape Verdean immigrants from higher socioeconomic backgrounds, constraining generalizability. Second, the participants’ perspectives on quality issues related to the U.S. mental health care system are, in part, influenced by experiences with mental health treatment, or lack thereof, in their native countries. An important future direction of this research is to explore questions regarding perceived quality of mental health care systems in the participants’ country of origin and previous experiences with mental health treatment in order to be able to contextualize their current perceptions on the quality of and experiences with the U.S. mental health care system.

Despite these limitations, this study is the first of its kind that examines QOC using CQR. This method allowed us to identify more nuanced quality-related factors that have theoretical and practical relevance, and not apparent from data collected using structured surveys and quantitative research methods (Cooper et al., 2003; Mulvaney-Day, Alegria, Earl, & Linhart, 2011). Study findings inform our understanding of patient-centered care across and within two understudied immigrant ethnic groups. Our findings provide useful information that mental health clinicians can use to increase their own cultural awareness and knowledge to foster productive patient-centered interactions and help improve engagement in mental health care with these immigrant groups. These findings are not meant to represent fixed prescriptions for clinical behavior, but rather to highlight the perspectives of these two immigrant groups. Tailoring mental health services to address the preferences of heterogeneous patient populations is a central tenant of patient-centered care, and has been shown to be associated with engagement in care, increased adherence, and improved outcomes of care (Lang & Shannon, 1997; Saha, Arbelaez, & Cooper, 2003; Sofaer & Firminger, 2005; Stichler & Weiss, 2000; Tam, 2007). Furthermore, these findings provide useful data for administrators and policymakers in the mental health care arena to develop more culturally appropriate and effective models and policies related to mental health care service delivery (Bettancourt et al., 2003). In particular, our findings suggest that improving aspects of mental health systems such as: (1) the provision of more professional development training opportunities, including on cross-cultural competence, for all mental health care staff and (2) the improvement of the structure in which care is delivered, including hospital buildings, financing, technology, and equipment are key components in QOC for these patient groups. These findings are also consistent with Donabedian's (1988) structure–process–outcome model for improving health care quality.

## Conclusion

The demographic changes that are anticipated over the next decade in the United States magnify the importance of addressing mental health care disparities. At the micro-level, the problem, in part, is related to the mismatch between patients’ and providers’ perspectives of quality health care provision. Compounding the problem for immigrants is the limited availability of bilingual clinicians and lack of knowledge about patients’ perspectives on what matters in a clinic encounter, potentially resulting in less accurate diagnoses and mismatches between treatment needs and resources. In order to provide quality care, knowledge of immigrant perspectives on what constitutes QOC mental health is not only informative but indeed necessary for clinicians, administrators, and policymakers. Future research is necessary in order to further examine immigrant patients’ perspectives on quality mental health care, while taking into account factors related to their prior experience with mental health care (or lack thereof) and the mental health system in their country of origin.

## References

[CIT0001] Alegria M, Canino G, Shrout P. E, Woo M, Duan N, Vila D (2008). Prevalence of mental illness in immigrant and non-immigrant U.S. Latino groups. American Journal of Psychiatry.

[CIT0002] Alegria M, Chatterji P, Wells K, Cao Z, Chen C, Takeuchi D (2008). Disparity in depression treatment among racial and ethnic minority populations in the United States. Psychiatric Services.

[CIT0003] American Community Survey (2006–2010). U.S. Census Bureau. http://www.census.gov/acs/www/.

[CIT0004] American Community Survey (2011). United States Census Bureau, U.S. Department of Commerce. http://www.census.gov/acs/www/about_the_survey/2011_acs_improvements/.

[CIT0005] Bettancourt J. R, Green A. R, Carrillo J. E, Ananeh-Firempong O (2003). Defining cultural competence: a practical framework for addressing racial/ethnic disparities in health and health care. Public Health Reports.

[CIT0006] Bowling A, Rowe G, Lambert N, Waddington M, Mahtani K. R, Kenten C (2012). The measurement of patients’ expectations for health care: A review and psychometric testing of a measure of patients’ expectations. Health Technol Asses.

[CIT0007] Browall M, Koinberg I, Falk H, Wijk H (2013). Patients’ experience of important factors in the healthcare environment in oncology care. International Journal of Qualitative Studies on Health and Wellbeing.

[CIT0008] Buck P. W, Alexander L. B (2006). Neglected voices: consumers with serious mental illness speak about intensive case management. Administration and Policy in Mental Health and Mental Health Services Research.

[CIT0009] Campbell S. M, Roland M. O, Buetow S. A (2000). Defining quality of care. Social Science and Medicine.

[CIT0010] Carling J (2002). Cape Verde: Towards the end of emigration?. Migration Policy Institute.

[CIT0011] Cooper L. A, Roter D. L, Johnson R. L, Ford D. E, Steinwachs D. M, Powe N. R (2003). Patient-centered communication, ratings of care, and concordance of patient and physician race. Annals of Internal Medicine.

[CIT0012] Donabedian A (1980). Explorations in quality assessment and monitoring. Vol. 1: The definition of quality and approaches to its assessment.

[CIT0013] Donabedian A (1988). The quality of care: How can it be assessed?. Journal of the American Medical Association.

[CIT0014] Earl T. R, Alegria M, Mendieta F, Linhart Y. D (2011). “Just be straight with me:” an exploration of Black patient experiences in initial mental health encounters. American Journal of Orthopsychiatry.

[CIT0015] Elliott R, Packer M. J, Addison R. B (1989). Comprehensive process analysis: Understanding the change process in significant therapy events. Entering the circle: Hermeneutic investigations in psychology.

[CIT0016] Flores G (2005). The impact of medical interpreter services on the quality of health care: A systematic review. Medical Care Research and Review.

[CIT0017] Giorgi A, Girogi A (1985). Sketch of a psychological phenomenological method. Phenomenology and psychological research.

[CIT0018] Grundberg A, Ebbeskog B, Dahlgren M. A, Religa D (2012). How community-dwelling seniors with multimorbidity conceive the concept of mental health and factors that may influence it: A phenomenographic study. International Journal of Qualitative Studies on Health and Wellbeing.

[CIT0019] Hill C. E, Thompson B. J, Williams E. N (1997). A guide to conducting consensual qualitative research. The Counseling Psychologist.

[CIT0020] Hill C. E, Knox S, Thompson B. J, Williams E. N, Hess S. A, Ladany N (2005). Consensual qualitative research: An update. Journal of Counseling Psychology.

[CIT0021] Institute of Medicine of the National Academies (2009). Race, ethnicity and language data: Standardization for health care quality improvement.

[CIT0022] Jun M, Peterson R. T, Zsidisin G. A (1998). The identification and measurement of quality dimensions in health care: Focus group interview results. Health Care Management Review.

[CIT0023] Kirsh B, Tate E (2006). Developing a comprehensive understanding of the working alliance in community mental health. Qualitative Health Research.

[CIT0024] Kitzinger J (1995). Qualitative research: Introducing focus groups. British Medical Journal.

[CIT0025] Lang L. A, Shannon T. E (1997). Value and choice: Providing consumers with information on the quality of health care. Conference overview. The Joint Commission Journal on Quality Improvement.

[CIT0026] Lima A, Siqueira C. E (2007). Brazilians in the U.S. and Massachusetts: A demographic and economic profile.

[CIT0027] Lohr K, Committee to Design a Strategy for Quality Review and Assurance in Medicare (1990). Medicare: a strategy for quality assurance.

[CIT0028] Martinsson G, Fagerberg I, Lindholm C, Wiklund-Gustin L (2012). Struggling for existence—Life situation experiences of older persons with mental disorders. International Journal of Qualitative Studies on Health and Wellbeing.

[CIT0029] Martinsson G, Wiklund-Gustin L, Lindholm C, Fagerberg I (2011). Being altruistically egoistic-Nursing aides’ experiences of caring for older persons with mental disorders. International Journal of Qualitative Studies on Health and Wellbeing.

[CIT0030] Miles M, Huberman A (1994). Qualitative data analysis.

[CIT0031] Mulvaney-Day N. E, Alegria M, Earl T, Linhart Y. D (2011). Preferences for relational style with mental health clinicians: a qualitative comparison of African American, Latino and non-Latino White patients. Journal of Clinical Psychology.

[CIT0032] Pope-Davis D. B, Toporek R. L, Ortega-Villalobos L, Ligiéro D. P, Brittan-Powell C. S, Liu W. M (2002). Client perspectives of multicultural counseling competence: A qualitative examination. The Counseling Psychologist.

[CIT0033] Pumariega A. J, Rothe E, Pumariega J. B (2005). Mental health of immigrants and refugees. Community Mental Health Journal.

[CIT0034] Rasmussen H, Hellzen O (2013). The meaning of long-term caregiving for patients with frontal lobe dementia. International Journal of Qualitative Studies on Health and Wellbeing.

[CIT0035] Saha S, Arbelaez J. J, Cooper L. A (2003). Patient-physician relationships and racial disparities in the quality of health care. American Journal of Public Health.

[CIT0036] Sixma H, Kerssens J, van Campen C, Peters L (1998). Quality of care from the patient's perspective: From theoretical concept to a new measuring instrument. Health Expectations.

[CIT0037] Smedley B. D, Stith A. Y, Nelson A. R (2003). Unequal treatment: Confronting racial and ethnic disparities in health care.

[CIT0038] Sofaer S, Firminger K (2005). Patient perceptions of the quality of health services. Annual Review of Public Health.

[CIT0039] Stichler J. F, Weiss M. E (2000). Through the eye of the beholder: Multiple perspectives on quality in women's health care. Quality Management in Health Care.

[CIT0040] Strauss A, Corbin J (1998). Basics of qualitative research: Grounded theory procedures and techniques.

[CIT0041] Tam J. L. M (2007). Linking quality improvement with patient satisfaction: A study of a health service centre. Marketing Intelligence & Planning.

[CIT0042] Tucker C. M, Herman K. C, Ferdinand L. A, Bailey T. R, Lopez M. T, Beato C (2007). Providing patient-centered culturally sensitive health care: a formative model. The Counseling Psychologist.

[CIT0043] Tunner T. P, Salzer M. S (2006). Consumer perspectives on quality of care in the treatment of schizophrenia. Administration and Policy in Mental Health and Mental Health Services Research.

[CIT0044] United States Census Bureau (2012). http://www.census.gov/compendia/statab/.

[CIT0045] Ware N. C, Tugenberg T, Dickey B (2004). Practitioner relationships and quality of care for low-income persons with serious mental illness. Psychiatric Services.

[CIT0046] Wills C. E, Holmes-Rovner M (2006). Integrating decision making and mental health interventions research: research directions. Clinical Psychology.

[CIT0047] Zayas L. H, Cabassa L. J, Perez M. C (2005). Capacity-to-consent in psychiatric research: development and preliminary testing of a screening tool. Research on Social Work Practice.

